# COVID-19: The Development and Validation of a New Mortality Risk Score

**DOI:** 10.3390/jcm13071832

**Published:** 2024-03-22

**Authors:** Giuseppe Zinna, Luca Pipitò, Claudia Colomba, Nicola Scichilone, Anna Licata, Mario Barbagallo, Antonio Russo, Piero Luigi Almasio, Nicola Coppola, Antonio Cascio

**Affiliations:** 1 Department of Health Promotion, Mother and Child Care, Internal Medicine and Medical Specialties, University of Palermo, 90127 Palermo, Italy; giuseppe.zinna@studenti.univr.it (G.Z.); luca.pipito@community.unipa.it (L.P.); claudia.colomba@unipa.it (C.C.); nicola.scichilone@unipa.it (N.S.); anna.licata@unipa.it (A.L.); mario.barbagallo@unipa.it (M.B.); piero.almasio@unipa.it (P.L.A.); 2 Department of Surgery, Dentistry, Paediatrics, and Gynaecology, Division of Cardiac Surgery, University of Verona Medical School, 37129 Verona, Italy; 3 Pediatric Infectious Disease Unit, ARNAS Civico-Di Cristina-Benfratelli Hospital, 90127 Palermo, Italy; 4 Section of Infectious Diseases, Department of Mental Health and Public Medicine, University of Campania “Luigi Vanvitelli”, Via Luciano Armanni 5, 80131 Naples, Italy; antonio.russo2@unicampania.it (A.R.); nicola.coppola@unicampania.it (N.C.); 5 Infectious and Tropical Disease Unit, AOU Policlinico “P. Giaccone”, Via del Vespro 129, 90127 Palermo, Italy

**Keywords:** COVID-19, SARS-CoV-2, hemoglobin, predictive model, risk score, internal validation, external validation

## Abstract

**Background:** The coronavirus disease 2019 (COVID-19) pandemic has found the whole world unprepared for its correct management. Italy was the first European country to experience the spread of the SARS-CoV-2 virus at the end of February 2020. As a result of hospital overcrowding, the quality of care delivered was not always optimal. A substantial number of patients admitted to non-ICU units could have been treated at home. It would have been extremely useful to have a score that, based on personal and clinical characteristics and simple blood tests, could have predicted with sufficient reliability the probability that a patient had or did not have a disease that could have led to their death. This study aims to develop a scoring system to identify which patients with COVID-19 are at high mortality risk upon hospital admission, to expedite and enhance clinical decision making. **Methods**: A retrospective analysis was performed to develop a multivariable prognostic prediction model. **Results**: Derivation and external validation cohorts were obtained from two Italian University Hospital databases, including 388 (10.31% deceased) and 1357 (7.68% deceased) patients with confirmed COVID-19, respectively. A multivariable logistic model was used to select seven variables associated with in-hospital death (age, baseline oxygen saturation, hemoglobin value, white blood cell count, percentage of neutrophils, platelet count, and creatinine value). Calibration and discrimination were satisfactory with a cumulative AUC for prediction mortality of 0.924 (95% CI: 0.893–0.944) in derivation cohorts and 0.808 (95% CI: 0.886–0.828) in external validation cohorts. The risk score obtained was compared with the ISARIC 4C Mortality Score, and with all the other most important scores considered so far, to evaluate the risk of death of patients with COVID-19. It performed better than all the above scores to evaluate the predictability of dying. Its sensitivity, specificity, and AUC were higher than the other COVID-19 scoring systems when the latter were calculated for the 388 patients in our derivation cohort. **Conclusions**: In conclusion, the CZ-COVID-19 Score may help all physicians by identifying those COVID-19 patients who require more attention to provide better therapeutic regimens or, on the contrary, by identifying those patients for whom hospitalization is not necessary and who could therefore be sent home without overcrowding healthcare facilities. We developed and validated a new risk score based on seven variables for upon-hospital admission of COVID-19 patients. It is very simple to calculate and performs better than all the other similar scores to evaluate the predictability of dying.

## 1. Introduction

Italy was the first European country to experience the spread of the SARS-CoV-2 virus at the end of February 2020, as well as one of the countries with the highest intra-hospital and extra-hospital mortality rates [1,2]. As a result of hospital overcrowding, the quality of care delivered has not always been optimal [3]. A substantial number of patients admitted to non-ICU units could have been treated at home [4]. It would have been extremely useful to have a score that, based on personal and clinical characteristics and simple blood chemistry tests, could have predicted with sufficient reliability the probability that a patient had or did not have a disease that could have led to their death [5]. Prediction models for coronavirus disease 2019 (COVID-19) have quickly entered the academic literature to support medical decision making at a time when they are urgently needed, but almost all published prediction models are poorly reported and at high risk of bias such that their reported predictive performance is probably optimistic [6]. Compared to adults hospitalized during early COVID-19 variant periods, those hospitalized during the Omicron variant were older, had multiple co-morbidities, were more likely to be vaccinated, and less likely to experience severe respiratory disease, systemic inflammation, coagulopathy, and death [7]. Currently, the ISARIC 4C mortality score is considered the most accredited prognostic model to identify patients with a high risk of progression, regardless of variant COVID-19; however, its calculation is not very easy and takes into consideration several parameters, some of which can lend themselves to evaluations subjective [8]. This study aims to develop a simple scoring system to identify which patients with COVID-19 are at high mortality risk upon hospital admission, expedite and enhance clinical decision making, and provide them with appropriate respiratory support and other treatments, such as monoclonal antibodies and/or antivirals, as soon as possible.

## 2. Materials and Methods

### 2.1. Study Design, Setting, and Study Population

This retrospective study was conducted at multiple departments of two Italian University hospitals during the first and second wave of the COVID-19 pandemic: the infectious and tropical disease unit, internal medicine unit, and geriatric unit of University Hospital “Policlinico Paolo Giaccone” of Palermo (Italy) and infectious and tropical disease unit of “Luigi Vanvitelli” University Hospital of Campania (Italy). First step: medical records of 388 consecutive patients admitted to the University Hospital of Palermo, Italy, during the first and second waves of the COVID-19 pandemic, between June 2020, beginning the first peak of COVID-19 in Sicily, and December 2021 were analyzed. These patients constituted the derivation cohort of the new prognostic score. Second step: medical records of 1357 consecutive patients admitted to “Luigi Vanvitelli” University Hospital of Campania (Italy) between June 2020 and December 2021 constituted the external validation cohort. Data collected refer to a period when treatment protocols were in their developing stage, and no standard treatment was available; therefore, none of the patients had previously received an “early therapy” with antivirals or monoclonal. Furthermore, in that period availability of hospital beds and oxygen were conditions that could have affected the risk of mortality. COVID-19 diagnosis was based on positive respiratory tract real-time polymerase chain reaction (RT-PCR) results for SARS-CoV-2 nucleic acid. Model development and reporting followed the TRIPOD (Transparent Reporting of a Multivariable Prediction Model for Individual Prediction or Diagnosis) guidelines [9]. This study was conducted in accordance with the Declaration of Helsinki. This study was approved by the Ethics Committee “Palermo I” of the A.O.U. Policlinico “Paolo Giaccone”, Palermo, Italy (verbal n. 11/2022, 12 December 2022).

### 2.2. Data Collection

For the derivation cohort, data about patients admitted to the University Hospital “Policlinico Paolo Giaccone” of Palermo (Italy), were extracted from paper-based medical records and collected in a Microsoft Access database. They included epidemiological and demographic characteristics, medical comorbidities, Charlson Comorbidity Index (CCI) on admission [10], days from onset of symptoms to hospitalization, days from positive swab to hospitalization, days from onset of symptoms to positive swab, National Early Warning Score (NEWS) on admission [11], Pneumonia Severity Index (PSI) on admission [12], clinical presentation, vital signs at first clinical contact (blood pressure, heart rate, oxygen saturation, respiratory rate, body temperature), respiratory function, laboratory tests, imaging, oxygen therapy, days of hospitalization, evolution during hospitalization and status at discharge. For the external validation cohort, patients admitted to the “Luigi Vanvitelli” University Hospital in Campania (Italy), the only variables on which our prognostic index was based, were extracted.

### 2.3. Statistical Analyses 

Data distribution was evaluated by the Shapiro–Wilk test. In descriptive analysis, mean and standard deviation (SD) were calculated for continuous values with normal distribution and median and interquartile range (IQR) for continuous values without normal distribution, and absolute and relative frequencies were calculated for categorical variables. In bivariate analysis, we calculated *p*-values using an unpaired Student’s *t* test or Mann–Whitney U test for continuous variables, and chi-squared for categorical variables. A *p*-value of less than 0.05 was considered statistically significant. All tests were 2-tailed. Multivariable analysis was performed using forward conditional stepwise logistic regression to examine the association between clinical and laboratory parameters and risk of death. The dependent variable was a favorable clinical outcome or death, and independent variables were selected according to clinical and statistical criteria in several stages. Crude odds ratios (OR) and their 95% CI for association of mortality with potential risk factors were calculated by univariate analysis. Adjusted OR (AOR) was calculated by stepwise logistic regression (LR) analysis to identify factors independently associated with mortality. For statistical analysis, we used the statistical package IBM SPSS, version 26; for graphical statistical presentation, we used GraphPad Prism Package, version 9, TIBCO Software Statistica—formerly StatSoft, version 10, and Microsoft Excel, version 2016.

#### 2.3.1. Development of a New Risk Scoring System 

For the derivation cohort, preliminary filtering of variables was performed by removing those that did not have statistical significance after bivariate analysis, had too many missing values (≥75% missing data), or were not collected immediately after hospitalization, e.g., days of hospitalization and imaging, which are usually performed later after hospital admission. Finally, we selected predictor variables based on the extent of multicollinearity and clinical relevance. A predictive model was developed based on seven covariates. The β and α parameters obtained from multivariable analysis were used to elaborate our prognostic index [13,14,15]. The predictive power of our final risk score to predict mortality risk was assessed by the receiver operating characteristic (ROC) curve and the corresponding area under the curve (AUC) with 95% confidence intervals (95% CI). The Hosmer–Lemeshow test was calculated for the logistic regression model. Finally, to validate the effectiveness of the prognostic score and the variables on which it is based, an internal validation was performed on the same derivation cohort, and an external validation on an external cohort.

#### 2.3.2. Performances Evaluation and Comparison

The CZ-COVID-19 score system was compared with the ISARIC 4C Mortality Score and, with all the other most important scores considered so far to evaluate the risk of death of patients with COVID-19, DeLong’s test was used to compare the CZ COVID-19 with the other scores calculated for our derivation cohort.

## 3. Results

### 3.1. Study Population, Univariate and Multivariable Analysis 

Detailed demographic and clinical data of 388 patients admitted at the University Hospital “Policlinico Paolo Giaccone” of Palermo (Italy) and 1357 patients admitted to the “Luigi Vanvitelli” University Hospital of Campania (Italy) are shown in Table 1 and Table S1. 

The percentages of alive and dead patients stratified by age group and sex are represented in Figure S1.

In Figure S2, the main comorbidities in patients who survived and who died are shown. The most common pathological background was arterial hypertension, with 207 (53.4%) cases, followed by diabetes (118, 30.4%) and cardiovascular disease (82, 21.1%). The five comorbidities, including other lung diseases (i.e., pneumonia, emphysema, pulmonary embolism, pulmonary fibrosis, primary or metastatic lung cancer, pneumoconiosis, etc.), chronic liver disease, cardiovascular disease, chronic kidney disease (CKD) and central nervous system (CNS) disease, were statistically significant in bivariate analysis but lost statistical significance in multivariable analysis associated with mortality (Table 1).

The CCI scores (median score 4 alive vs. 5 dead, *p* < 0.001), NESW (median score 3 alive vs. 6 dead, *p* < 0.001) and PSI (median score 2 alive vs. 4 dead, *p* < 0.001) were significantly higher in patients who died (Table 1). The threshold values of scores identified by the ROC curves for whom the risk of mortality was greater were CCI > 4 (crude OR 4.42, 95% CI 2.14–9.15), NEWS ≥ 7 (crude OR 8.29, 95% CI 3.40–20.20) and PSI ≥ III (crude OR 43.36, 95% CI 6.30–341.22), respectively (Table S1). In the multivariable model, only the PSI score retained statistical significance (AOR 19.33, 95% CI 1.18–317.77, *p*: 0.038) (Table S1).

The median duration from onset of symptoms to hospital admission was 6, IQR 3–10, days (4 days, IQR 1–8, in dead, *p*: 0.025). The median duration from symptom onset to positive SARS-CoV-2 molecular test was 3, IQR 0–7, days (2 days, IQR 0–3, in dead, *p*: 0.034). The median duration of hospitalization was 12, IQR 8–17, days (19 days, IQR 13–29, in dead, *p*: 0.002) (Table 1).

**Figure 1:**
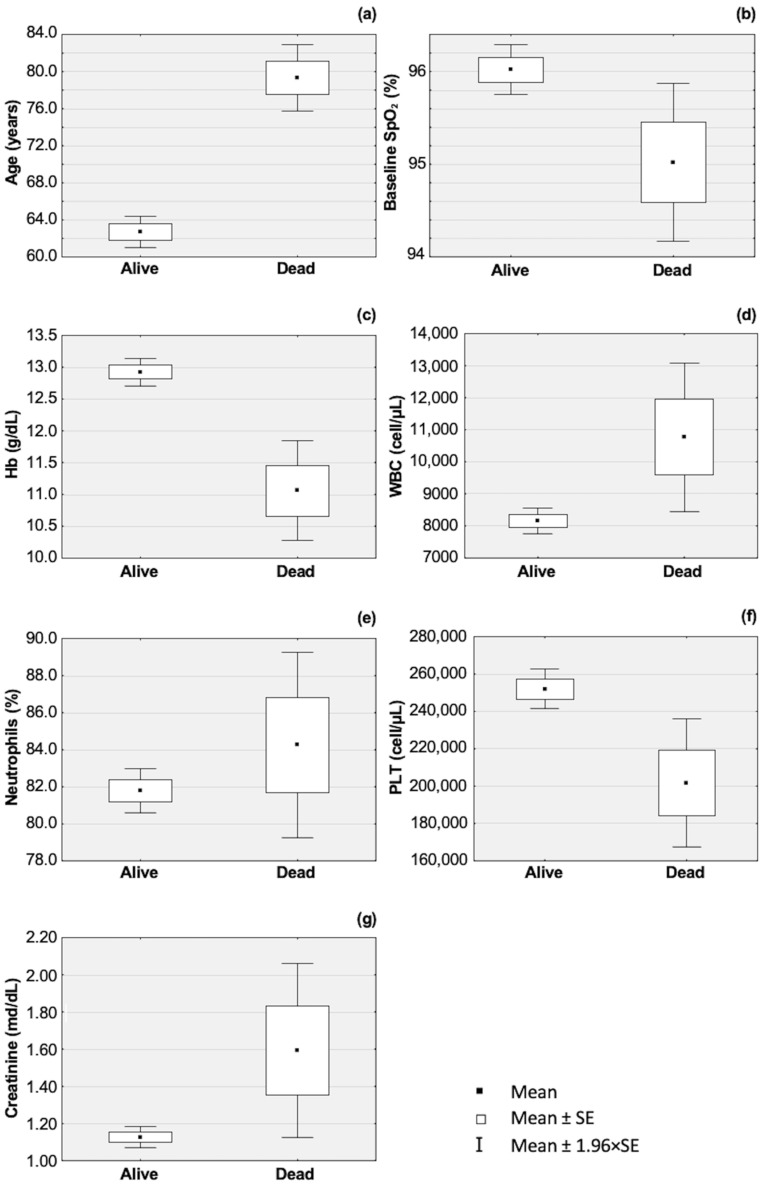
Box and whisker plots of the seven parameters included in the LR for the CZ-COVID-19 Score in surviving and deceased patients. Age (**a**), baseline SpO_2_ by pulse oximetry (**b**), Hb value (**c**), WBC count (**d**), percentage of neutrophils (**e**), PLT count (**f**), and creatinine value (**g**) at admission in surviving and deceased patients..

Hemoglobin (Hb) low values and platelet (PLT) low counts were frequently found in dead patients. Mean ± SD Hb values were 11.0 ± 2.5 g/dL (*p*: <0.001) in deceased derivation cohort patients, and 13.4 ± 1.1 g/dL (*p*: 0.05) in deceased external validation cohort patients, respectively (Table 1). Mean ± SD PLT count was 202 ± 111 × 10^3^ cell/µL (*p*: 0.004) in deceased derivation cohort patients, and 305 ± 107 × 10^3^ cell/µL (*p*: 0.174) in deceased external validation cohort patients, respectively (Table 1). The other way around, white blood cell (WBC) count, neutrophil count, and creatinine value were significantly higher at admission in patients who died. The Mean ± SD WBC was 10,765 ± 7476 cell/µL (*p*: <0.001) in deceased derivation cohort patients, and 9723 ± 5008 cell/µL (*p*: 0.006) in deceased external validation cohort patients, respectively (Table 1). The Mean ± SD neutrophil count was 9223 ± 6679 cell/µL (*p*: <0.001) in deceased derivation cohort patients, and 8201 ± 4743 cell/µL (*p*: 0.001) in deceased external validation cohort patients, respectively (Table 1). The Mean ± SD creatinine value was 1.75 ± 1.81 mg/dL (*p*: <0.001) in deceased derivation cohort patients, and 1.32 ± 1.00 mg/dL (*p*: <0.001) in deceased external validation cohort patients, respectively (Table 1). The above laboratory parameters showed statistical significance in both bivariate and multivariable analyses. The box graphs in Figure 1 show the seven parameters included in the LR model.

Higher acute phase reactants at admission were found in the deceased patients, although the difference was significant only for the ferritin values.

Mean ± SD CRP (48.9 ± 57.9 mg/L alive vs. 60.8 ± 63.6 mg/L dead, *p*: 0.275), Mean ± SD IL-6 (47.2 ± 174.3 pg/mL alive vs. 54.5 ± 55.5 pg/mL dead, *p*: 0.842), Mean ± SD ferritin (663 ± 705 ng/mL vs. 1263 ± 1184 ng/mL dead, *p*: 0.012) and Mean ± SD D-Dimer (2647 ± 7876 ng/mL EFU vs. 3763 ± 6987 ng/mL EFU dead, *p*: 0.463) (Table 1).

Baseline oxygen saturation (SpO_2_ %) by pulse oximetry was lower in deceased patients (95 ± 3%, *p*: 0.013). Two respiratory function parameters (breaths/minute and pH) were different in the bivariate statistical analysis; however, they could not be included in the LR model due to missing data (Table 1 and Table S1). In total, 220 (56.7%) patients did not require oxygen therapy upon admission; of this 220 patients group, 19 (40.0%) subsequently died. Remaining patients who later died had at admission, due to desaturation 7 (17.5%) had required nasal cannulas, 4 (10.0%) had required facial masks, 9 (22.5%) had required venture masks and only 1 (2.1%) had required invasive mechanical ventilation since admission (IMV) (Table 1).

A total of 40 patients (10.31%) died, 19 males (47.5%) and 21 females (52.5%) in the derivation cohort, and 104 patients (7.68%) died, 56 males (53.8%) and 48 females (46.2%) in the external validation cohort (Table 1). Of the 348 patients who survived, 261 (67.3%) were discharged home, 65 (16.8%) were transferred to a COVID-19 hotel, and 22 (5.7%) were transferred to other wards (Table 1).

The final multivariable model for predicting death is shown in Table 2. It includes only seven variables: age (years), baseline SpO_2_ by pulse oximetry (%), Hb value (g/dL), WBC count (cell/µL), neutrophils (%), PLT count (cell/µL), and creatinine value (mg/dL). 

**Figure 2:**
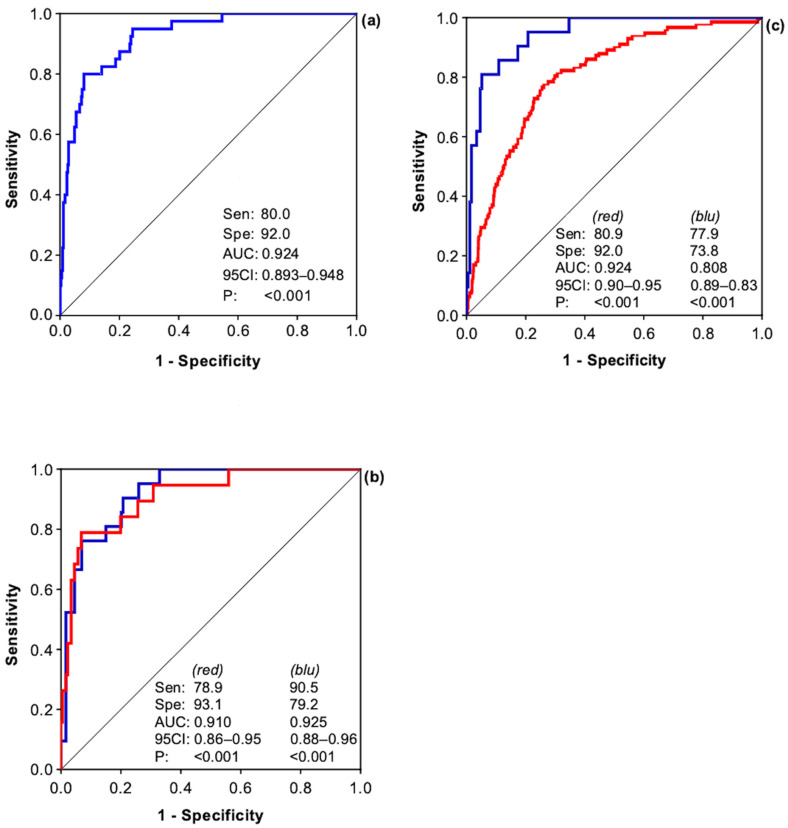
ROC curves of the CZ-COVID-19 Scores, over the total sample of 388 patients (**a**); comparison of the ROC curves between the two sub-cohorts used for internal validation (**b**); comparison of the ROC curves between the derivation cohort in blue and the external validation cohort in red (**c**). Sen: sensitivity; Spe: specificity; AUC: area under the curve; 95CI: 95% confidence interval; P: *p*-value.

Statistical analysis of the ROC curve of the derivation cohort showed an AUC of 0.924 (95% CI 0.893–0.948), sensitivity of 80.0%, and a specificity of 92.0%. A level of significance *p* < 0.001 for a CZ-COVID-19 Score that has a cut point ≤ 1 (Figure 2a). To internally validate the effectiveness of the prognostic score and the variables on which it is based, the sample of 388 patients was randomly divided into two equal cohorts of 194 patients: the first cohort was used to derive the prognostic score (cohort 1) and the second cohort was used for internal validation (control cohort). The comparative analysis of the ROC curves underlines the high sensitivity of the score for both cohorts (Figure 2b). To externally validate the effectiveness of the prognostic score and the variables on which it is based, an external validation cohort of 1357 patients admitted to the “Luigi Vanvitelli” University Hospital of Campania (Italy) was used. The comparative analysis of the ROC curves underlines the high sensitivity of the score for both cohorts, with an AUC in the external validation cohort of 0.808 (95% CI 0.886–0.828), and with a sensitivity and specificity of 77.9% and 73.8%, respectively (Figure 2c). 

The LR requirements were met. Of the remaining factors that might have independent prognostic value, only PSI, CCI, mute past medical history, other lung diseases, chronic liver disease, cardiovascular disease, CKD, diseases of the CNS, other comorbidities, days from symptom onset to hospital admission, days from symptom onset to positive swab, and cough at clinical onset were statistically significant in univariate statistical analysis. However, these did not improve the overall performance of the multivariable model. We did not evaluate ferritin, respiratory rate, and pH in the multivariable analysis because of many missing data at hospital admission (Table 1).

### 3.2. Prognostic Model COVID-19 Mortality

The developed prognostic model is based on seven covariates, one demographic variable, and six clinical and laboratory findings: age (years), baseline SpO_2_ by pulse oximetry (%), Hb (g/dL), WBC count (cell/µL), neutrophils (%), PLT (cell/µL), and creatinine value (mg/dL). The obtained score will be called the “Cascio-Zinna COVID-19-mortality Score” (CZ-COVID-19 Score). Its formula is

CZ-COVID-19 Score = −[26.084 + (0.166 × age (years)) + (−0.175 × baseline SpO_2_ (percentage)) + (−0.459 × Hb (g/dL) + (1.935 × In [WBC (cell/μL)]) + (−0.045 × neutrophils (percentage)) + (−2.327 × In [PLT (cell/μL)]) + (1.108 × In [creatine (mg/dL)])]

Probability of death = σ (r)

where age, baseline SpO_2_ % by pulse oximetry, Hb value, WBC count, neutrophils %, PLT count, and creatinine value are input predictors of the LR model, r is the risk score, and σ is the sigmoid function; that is,

σ (CZ-COVID-19 Score) = 1/(1 + e^−(CV-COVID−19 Score)^)

for the LR, the natural logarithm of WBC count, the natural logarithm of PLT count, and the natural logarithm of creatinine value were used, with a value of 1 for each parameter less than one (i.e., if creatinine is 0.7, a value of 1.0 was used) to prevent the natural logarithm of the positive number less than 1 (greater than 0 and less than 1) from yielding a negative value. The model achieved a cumulative AUC value of more than 92.4% in the derivation cohort (Figure 2a), and 80.8% in the external validation cohort (Figure 2c). The Hosmer–Lemeshow test for Pearson’s chi-squared goodness of fit equals 0.992 in the derivation cohort and 0.306 in the external validation cohort.

The risk of death for each patient was calculated at the time of admission. Patients were divided into three groups according to their scores: (1) a low-risk group (score ≥ 4) (219 patients in the derivation cohort, 56.44% of the sample vs. 272 patients in the external validation cohort, 20.04% of the sample); (2) an intermediate-risk group (score 2–3) (105 patients in derivation cohort, 27.06% of the sample vs. 245 patients in external validation cohort, 18.05% of the sample); (3) a high-risk group (score ≤ 1) (64 patients in derivation cohort, 16.49% of the sample vs. 840 patients in external validation cohort, 61.90% of the sample). The threshold values of ≥4, 2–3, and ≤1 of the CZ-COVID-19 Score identify with a specificity and a sensitivity of 92% and 80%, respectively, the probability of dying in 5%, 15%, and 80% (*p*: <0.001 by Log-rank test) (Table S2).

Finally, we compared the prognosis of each study cohort among low vs. intermediate-risk individuals (*p*-value = 0.005), intermediate vs. high-risk individuals (*p*-value < 0.001), and low vs. high-risk individuals (*p*-value < 0.001). To facilitate the application of the model in a clinical setting, Table S2 includes lookup tables to calculate a patient’s death probability.

**Figure 3:**
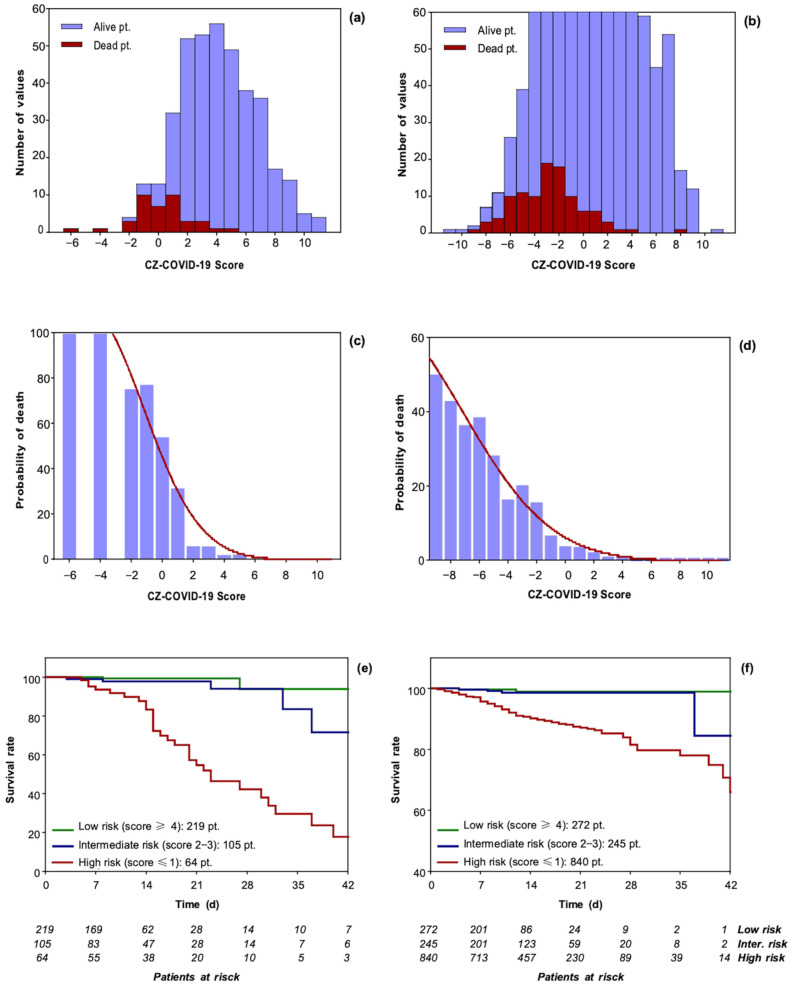
Distributions of CZ-COVID-19 Score in patients (pt.) for alive and dead patients for derivation cohort (**a**) and external validation cohort (**b**). Probability of death as a function of CZ-COVID-19 Score for derivation cohort (**c**) and external validation cohort (**d**), the model (red curve) almost perfectly follows the probability of death (blue bars) calculated directly from the data. Kaplan–Meier survival curves for derivation cohort (**e**) external validation cohort (**f**), of the low, intermediate, and high-risk patients. The threshold values of ≥4, 2–3, and ≤1 of the CZ-COVID-19 Score identify with a specificity and a sensitivity of 92% and 80%, respectively, the probability of dying in 5%, 15%, and 80% (*p*: <0.001 by Log-rank test).

Distributions of the CZ-COVID-19 Score for surviving and deceased patients are shown in Figure 3a,b, and the probability of death is shown in Figure 3c,d. A physician can easily calculate the probability of death; the higher the score, the greater the probability that the patient will survive. Figure 3e,f shows the Kaplan–Meier curves of low, intermediate, and high-risk groups in the two patient cohorts.

Using Excel, we developed an application algorithm that could be easily used on PCs and mobile phones (Supplementary Materials, Spreadsheet A: CZ-COVID-19 Score calculator).

### 3.3. Comparison with Other Standard Scores

The results of the comparative analysis of the ROC curves of our CZ-COVID-19 Score versus other COVID-19 scoring systems for the 388 patients in the derivation cohort are shown in Table 3. The CZ-COVID-19 Score with its AUC = 0.924, CI 95% 0.893–0.948 performed better than all the following other scores considered: Charlson Comorbidity Index (CCI) [10]; Pneumonia Severity Index (PSI) [12]; National Early Warning Score (NEWS) on admission [11]; International Severe Acute Respiratory Infection Consortium–Coronavirus Clinical Characterisation Consortium (ISARIC 4C) [16]; Hospitalisation or Outpatient Management of Patients with SARS-CoV-2 (HOME-CoV) [17]; ABC2-SPH risk score (ABC2-SPH) [18]; CAPS-D score [19]; SOARS score abbreviated for SpO_2_, obesity, age, respiratory rate, stroke history (SOARS) [20], COVID-19 severity index [21], ASCL score abbreviated for age, sex, CRP at hospital admission, and LDH at hospital admission (ASCL) [22]; COVID-19 Early Warning Score (COEWS) [23]; National Early Warning Score 2 Plus (NEWS 2 Plus) [24] (Table 3).

## 4. Discussion

Our analysis shows a strong association between mortality from COVID-19 and advanced age, low Hb value, low PLT count, and low SpO_2_ %, an increase in WBC count, neutrophil %, and creatinine value measured at the time of hospitalization. 

Observations from our single-center retrospective study showed that SARS-CoV-2 patients with Hb levels lower than 12.1 g/dL are at higher risk of in-hospital mortality than those with higher levels. Anemia has been reported in the course of pneumonia, ranging its prevalence between 7% and 15%. Hb values of 10 g/dL or less have been associated with an increase in 90 d mortality in hospitalized patients with community-acquired pneumonia or with COVID-19 [25,26,27]. The Hb concentration level influences arterial oxygen content and low Hb levels can lead to impairment of tissue oxygen delivery [28]. Physiologically, anemic hypoxia induces general vasodilation, but also pulmonary vasoconstriction, with an increase in fibrin formation in lung microvasculature [29]. Indeed, patients with similar SpO_2_ % were more likely to die when their Hb decreased below 12.1 g/dL [30]. SARS-CoV-2-related infection can also impair iron metabolism and reduce iron availability [31]. Furthermore, virus-induced intestinal mucosal erosion in SARS-CoV-2 and associated bleeding have been reported [32], and elderly hospitalized SARS-CoV-2 patients were found to have lower Hb levels [33]. Anemia in SARS-CoV-2 infection may be related to cytokine-induced inhibition of erythropoietin synthesis and may lead to the increased need for mechanical ventilation [34]. A systematic review of 63 studies showed that severe SARS-CoV-2 infection is associated with lower Hb levels [35], and a study by Fan et al. showed that 1.6% of SARS-CoV-2 patients admitted to the intensive care unit received blood transfusions for anemia correction [36]. Most studies, including ours, have shown that anemia is an independent predictor of mortality and that each unit of increase in Hb in COVID-19 patients enhanced the survival rate by 4% [30,37,38]. A meta-analysis based on risk factor-adjusted effect estimates indicated that anemia was independently associated with a significantly elevated risk for mortality among COVID-19 patients [39], while another study documented that in COVID-19 patients, anemia is both associated with a more pronounced baseline pro-inflammatory profile and a higher incidence of in-hospital mortality and severe disease [40].

Several studies focus on COVID-19 patients’ increased thromboembolic risk. Protein C could play a mechanistic role in the hypercoagulability syndrome affecting patients with severe COVID-19 [41]. The overwhelming inflammatory response in patients with SARS-CoV-2 infection can lead to a hypercoagulable state [42]. It seems that the increase in acute phase reactants (CRP, IL-6, ferritin, and D-Dimer) reflects the inflammation caused by SARS-CoV-2, but also the consumption of coagulation factors [43]. In our cohort, such an association was not observed for laboratory tests executed at the beginning of hospitalization. All variables associated with coagulation were measured once at the beginning of hospitalization, and we cannot exclude their changes over time. Still, although outside the compass of this study, analysis of laboratory parameters during the hospital stay at a 16-day follow-up reveals a reduction of natural plasma anticoagulants, in agreement with what has been reported above.

The most popular models that combine comorbidity and severity with mortality data in patients with pneumonia and COVID-19 are CCI, PSI, NEWS, ISARIC 4C, HOME-CoV, ABC2-SPH, CAPS-D, SOARS, COVID-19 severity index, ASCL, COEWS, and NEWS 2 Plus. The calculation of the PSI is very complex and includes a large number of variables. Some variables may not be easily available and lend themselves to human error [12]. The ISARIC 4C Mortality Score, developed in the UK in 2020, outperformed existing scores and showed utility in directly impacting clinical decision making [16,44]. Several studies evaluate the dynamic use of the ISARIC 4C score at admission, using AUC to assess score discrimination on admission. In COVID-19 patients, Crocker-Buque et al. [45] obtained an AUC of 0.71 (95% CI: 0.69–0.74), while Jones et al. in a validation study, obtained an AUC of 0.77 (95 CI 0.79–0.87) at admission [45,46]. These AUCs appear to perform less well than the one obtained with our CZ-COVID-19 Score on admission. Furthermore, the calculation of the ISARIC 4C requires more time and many more parameters, which are not very easy to calculate. For example, for its calculation, it is necessary to evaluate the Glasgow Coma Scale and the presence of comorbidities whose presence or absence is established by a healthcare professional (it is not objective data). It can be noted that our developed score system, unlike other scores, is based on objective variables that can be easily and quickly determined in all hospital settings.

Even though nowadays management protocols are better defined, and intensive care facilities are experimenting optimally to reduce mortality, our score identifies hidden high-risk patients and therefore deserving of greater therapeutic attention based only on objective variables that are easily measurable in any laboratory. Our score in our patients, as shown in the ROC curves described in Table 3, performed much better than the ISARIC 4C mortality score and all other scores tested.

Most clinical studies focused on patients admitted to the Intensive Care Unit (ICU) [47,48], our study is among the very few that treat COVID-19 patients hospitalized not in intensive care with the aim of identifying independent predictors of mortality [49]. Combining blood test results with simple clinic demographic data allows patients to be stratified based on mortality risk. Such stratification is essential to recognize non-critical patients who are more likely to die from COVID-19 and who therefore require greater assistance and care [49,50].

Although in several clinical studies, researchers have concentrated on patients with comorbidities like arterial hypertension, diabetes, chronic liver disease cardiovascular disease, chronic kidney disease, and disease of the central nervous system, emphasizing a significant rise in comorbidities and a more aggressive course of the disease necessitating a higher rate of ICU admission and oxygen use [51,52,53,54,55,56], in our retrospective cohort, arterial hypertension and diabetes were not found to be associated with an increase in mortality.

## 5. Conclusions

The CZ-COVID-19 Score we developed reliably predicts hospitalized patients’ risk of dying from COVID-19 and has three main advantages: (1) it is based on seven variables (age, baseline SpO_2_ %, Hb value, WBC count, neutrophils %, PLT count, and creatinine value) that are objective and always available for all patients hospitalized or admitted to an emergency department for COVID-19; (2) it is easy to calculate thanks to the Excel application found in the supplementary material; and (3) it performed better than all the other scores described before to evaluate the predictability of dying of our patients. Its sensitivity, specificity, and AUC were higher than the other COVID-19 scoring systems. We hope that in the future our score can be used all over the world and its effectiveness can be further validated.

Our score may identify hidden high-risk patients early enough to provide them with appropriate respiratory support and other treatments such as monoclonal antibodies and/or antivirals as soon as possible. Furthermore, the model provides a continuous probability of death instead of classifying risks based on thresholds, as in previous studies.

Our score can be used as a simple tool for non-specialist physicians to classify the severity of the disease in the early stages of patients with COVID-19. In conclusion, the CZ-COVID-19 Score may help all physicians by identifying those COVID-19 patients who require more attention to provide better therapeutic regimens or, on the contrary, by identifying those patients for whom hospitalization is not necessary and who could therefore be sent home without overcrowding healthcare facilities.

## Figures and Tables

**Table 1:** Characteristics of the 388 patients of the derivation and the 1357 patients of the external validation cohorts. Demographics, comorbidities, calculated scores on admission to hospital, time passed, symptoms and signs at clinical onset, laboratory test, respiratory function, imaging, hospital oxygen therapy by clinical status on admission, and clinical outcome. Bivariate analysis according to status at discharge. For each variable, the total number of valid data is given in the first column. For each outcome, the absolute frequency, the percentage of the outcome, and the *p*-values for the comparison between alive and dead at discharge are given. Statistically significant associations are shown in bold. SD: standard deviation; IQR: interquartile range; COPD: chronic obstructive pulmonary disease; TB: tuberculosis; IV: intravenous therapy; HIV: human immunodeficiency virus; CKD: chronic kidney disease; AKI: acute kidney injury; CNS: central nervous system; CCI: Charlson Comorbidity Index; IQR: interquartile range; PSI: Pneumonia Severity Index; NEWS: National Early Warning Score; Hb: hemoglobin; WBC: white blood cells; PLT: platelets; LDH: lactate dehydrogenase; PT/INR: Prothrombin Time/International Normalized Ratio; aPTT: activated partial thromboplastin time; CRP: C-reactive protein; PCT: procalcitonin; IL-6: interleukin-6; BNPT: B-type natriuretic peptide; HR: heart rate; SpO_2_: oxygen saturation; Pa: arterial partial pressure; FiO_2_: fraction of inspired O_2_; PaO_2_St: standardized arterial partial pressure oxygen; NIV: non-invasive ventilation; IMV: invasive mechanical ventilation; (*) data referring to the external validation cohort are highlighted with an asterisk and in italic.

Variables		Vital Status at Discharge		*p* Value
	All Patients	Alive	Dead	
Derivation Cohort Validation Cohort *	*n*: 388 n: 1357 *	*n*: 348 n: 1253 *	*n*: 40 n: 104 *	
**Demographics**				
Age (mean ± SD) Age (*n*: 388) Age (n: 1357) *	64.4 ± 16.5 *61.3 ± 16.1*	62.7 ± 16.1 *60.0 ± 15.5*	79.4 ± 11.5 *78.4 ± 12.8*	<0.001 *0.001*
Sex (*n*: 388)				
Male (%)	231 (59.5%)	212 (60.9%)	19 (47.5%)	0.101
Female (%)	157 (40.5%)	136 (39.1%)	21 (52.5%)	
Sex (n: 1357) *				
Male (%) *	*826 (60.7%)*	*770 (61.5%)*	*56 (53.8%)*	*0.127*
Female (%) *	*531 (39.0%)*	*483 (38.5%)*	*48 (46.2%)*	
**Comorbidities (%)**				
Mute past medical history (*n*: 388)	39 (10.1%)	39 (11.1%)	0 (0%)	0.026
Obesity (*n*: 388)	31 (8.0%)	29 (8.3%)	2 (5.0%)	0.462
Diabetes (*n*: 388)	118 (30.4%)	105 (30.2%)	13 (32.5%)	0.762
Hypertension (*n*: 388)	207 (53.4%)	182 (52.3%)	25 (65.5%)	0.221
COPD (*n*: 388)	29 (7.5%)	24 (6.9%)	5 (12.5%)	0.202
Asthma (*n*: 388)	6 (1.5%)	6 (1.7%)	0 (0%)	0.403
TB (*n*: 388)	3 (0.8%)	2 (0.6%)	0 (0%)	0.188
Other lung diseases (*n*: 388)	12 (5.4%)	16 (4.6%)	5 (12.5%)	0.036
Active smoking (*n*: 388)	13 (3.4%)	11 (3.2%)	2 (5.0%)	0.540
IV Drugs (*n*: 388)	1 (0.3%)	1 (0.3%)	0 (0%)	0.734
Alcoholism (*n*: 388)	4 (1.0%)	4 (1.1%)	0 (0%)	0.496
Depression (*n*: 388)	17 (4.4%)	17 (4.9%)	0 (0%)	0.153
Suicidal ideation (*n*: 388)	1 (0.3%)	1 (0.3%)	0 (0%)	0.734
Hepatosplenomegaly (*n*: 388)	3 (0.8%)	2 (0.6%)	1 (2.5%)	0.188
Gastrointestinal bleeding (*n*: 388)	2 (0.5%)	2 (0.6%)	0 (0%)	0.631
Hyperglycemia (*n*: 388)	58 (14.9%)	51 (14.7%)	7 (17.5%)	0.633
HIV+ (*n*: 388)	3 (0.8%)	2 (0.6%)	1 (2.5%)	0.188
Chronic liver disease (*n*: 388)	22 (5.7%)	14 (4.0%)	8 (20.0%)	<0.001
Cardiovascular disease (*n*: 388)	82 (21.1%)	66 (19.0%)	16 (40.0%)	0.002
Other heart conditions (*n*: 388)	61 (15.7%)	52 (14.9%)	9 (22.5%)	0.214
CKD (*n*: 388)	32 (8.2%)	24 (6.9%)	8 (20.0%)	0.004
AKI (*n*: 388)	5 (1.3%)	4 (1.1%)	1 (2.5%)	0.473
Hemodialysis (*n*: 388)	1 (0.3%)	1 (0.3%)	0 (0%)	0.734
Diseases of the CNS (*n*: 388)	39 (10.1%)	29 (8.3%)	10 (25.0%)	0.001
Organ transplant (*n*: 388)	5 (1.3%)	5 (1.4%)	0 (0%)	0.445
Other comorbidities (*n*: 388)	253 (65.2%)	221 (63.5%)	32 (80.0%)	0.038
**Scores (median IQR)**				
CCI (*n*:388)	4 (2–6)	4 (2–5)	5 (4–7)	<0.001
NEWS (*n*: 284)	3 (1–5)	3 (1–5)	6 (3–8)	<0.001
PSI (*n*: 388)	3 (2–3)	2 (2–3)	4 (3–5)	<0.001
**Time passed (median IQR)**				
Days of hospitalization (*n*: 386) Days of hospitalization (n: 1357) *	12 (8–18) *16 (15–16)*	12 (8–17) *16 (16–18)*	19 (13–29) *12 (11–14)*	0.002 *0.003*
Days from symptom onset to hospitalization (*n*: 309)	6 (3–10)	7 (3–10)	4 (1–8)	0.025
Days from positive swab to hospitalization (*n*: 360)	1 (0–3)	1 (0–3)	1 (0–2)	0.521
Days from symptom onset to positive swab (*n*: 302)	3 (0–7)	4 (0–7)	2 (0–3)	0.034
**Symptoms clinical onset (%)**				
Fever (*n*: 388)	201 (51.8%)	183 (52.6%)	18 (45.0%)	0.363
Cough (*n*: 388)	95 (24.2%)	93 (26.7%)	2 (5.0%)	0.002
Sputum (*n*: 388)	4 (1.0%)	3 (0.9%)	1 (2.5%)	0.331
Asthenia (*n*: 388)	71 (18.3%)	64 (18.4)	7 (17.5%)	0.890
Dyspnoea (*n*: 388)	144 (36.9%)	129 (37.1%)	15 (37.5%)	0.957
Anorexia (*n*: 388)	4 (1.0%)	4 (1.1%)	0 (0%)	0.496
Myalgia (*n*: 388)	24 (6.2%)	22 (6.3%)	2 (5.0%)	0.742
Arthalgia (*n*: 388)	25 (6.4%)	24 (6.9%)	1 (2.5%)	0.283
Loss of smell (*n*: 388)	13 (3.4%)	13 (3.7%)	0 (0%)	0.214
Loss of taste (*n*: 388)	13 (3.4%)	13 (3.7%)	0 (0%)	0.214
Diarrhea (*n*: 388)	34 (8.8%)	33 (9.5%)	1 (2.5%)	0.139
Vomit (*n*: 388)	18 (4.6%)	16 (4.6%)	2 (5.0%)	0.909
Headache (*n*: 388)	27 (7.0%)	27 (7.8%)	0 (0%)	0.068
Chest pain (*n*: 388)	19 (4.9%)	18 (5.2%)	1 (2.5%)	0.458
Abdominal pain (*n*: 388)	22 (5.7%)	22 (6.3%)	0 (0%)	0.102
Gastrointestinal bleeding (*n*: 388)	2 (0.5%)	2 (0.6%)	0 (0%)	0.631
Other symptoms (*n*: 388)	88 (22.7%)	77 (22.1%)	11 (27.5%)	0.448
**Laboratory test (mean ± SD)**				
Hb (g/dL) (*n*: 386) Hb (g/dL) (n: 1357) *	12.7 ± 2.2 *13.7 ± 1.3*	12.9 ± 2.1 *13.7 ± 1.3*	11.0 ± 2.5 *13.4 ± 1.1*	<0.001 *0.050*
WBC (cell/µL) (*n*: 387) WBC (cell/µL) (n: 1357) *	8418 ± 4506 *8700 ± 3971*	8146 ± 3955 *8615 ± 3863*	10,765 ± 7476 *9723 ± 5008*	<0.001 *0.006*
Number of neutrophils (cell/µL) (*n*: 381) Number of neutrophils (cell/µL) (n: 1357) *	7067 ± 4263 *7071 ± 3668*	6827 ± 3847 *6977 ± 3551*	9223 ± 6679 *8201 ± 4743*	<0.001 *0.001*
Percentage of neutrophils (%) (*n*: 381) Percentage of neutrophils (%) (n: 1357) *	82.1 ± 12.0 *79.3 ± 11.5*	81.9 ± 11.4 *79.0 ± 11.4*	84.3 ± 16.6 *82.9 ± 12.3*	0.258 *0.001*
Number of lymphocytes (cell/µL) (*n*: 381) Number of lymphocytes (cell/µL) (n: 1357) *	1307 ± 1071 *1049 ± 904*	1328 ± 1099 *1051 ± 834*	1112 ± 755 *1023 ± 1514*	0.239 *0.762*
Percentage of lymphocytes (%) (*n*: 381) Percentage of lymphocytes (%) (n: 1357) *	17.9 ± 12.0 *13.8 ± 9.6*	18.1 ± 11.4 *14.0 ± 9.4*	15.8 ± 16.5 *11.3 ± 10.8*	0.258 *0.005*
PLT (×10^3^/µL) (*n*: 383) PLT (×10^3^/µL) (n: 1357) *	248 ± 104 *291 ± 110*	252 ± 103 *290 ± 111*	202 ± 111 *305 ± 107*	0.004 *0.174*
Creatinine (mg/dL) (*n*: 388) Creatinine (mg/dL) (n: 1357) *	1.06 ± 0.86 *0.99 ± 0.86*	0.99 ± 0.67 *0.96 ± 0.83*	1.75 ± 1.81 *1.32 ± 1.00*	<0.001 *<0.001*
LDH (U/L) (*n*: 261)	268 ± 108	265 ± 104	294 ± 137	0.239
PT/INR (*n*: 356)	1.78 ± 7.72	1.80 ± 8.09	1.64 ± 1.77	0.910
aPTT (seconds) (*n*: 246)	29.6 ± 14.8	29.5 ± 14.7	30.9 ± 15.7	0.691
Fibrinogen (mg/dL) (*n*: 308)	522 ± 187	522 ± 182	516 ± 236	0.879
D-Dimer (ng/mL EFU) (*n*: 319)	2749 ± 7796	2647 ± 7876	3763 ± 6987	0.463
CRP (mg/L) (*n*: 356)	50.0 ± 58.4	48.9 ± 57.9	60.8 ± 63.6	0.275
PCT (µg/L) (*n*: 196)	1.4 ± 8.4	1.5 ± 9.0	0.5 ± 0.6	0.571
IL-6 (pg/mL) (*n*: 232)	47.9 ± 166.3	47.2 ± 174.3	54.5 ± 55.5	0.842
Triglycerides (mg/dL) (*n*: 97)	133 ± 62	134 ± 65	125 ± 26	0.677
Ferritin (ng/mL) (*n*: 111)	729 ± 786	663 ± 705	1263 ± 1184	0.012
Troponin (mg/L) (*n*: 56)	163 ± 674	180 ± 712	15 ± 16	0.576
BNPT (pg/mL) (*n*: 58)	1265 ± 2289	1377 ± 2408	444 ± 748	0.316
**Respiratory function (mean ± SD)**				
Acts breath/minute (*n*: 140)	18 ± 5	18 ± 5	21 ± 6	0.014
HR (*n*: 287)	83 ± 14	83 ± 14	84 ± 13	0.735
Baseline SpO_2_ (*n*: 361) Baseline SpO_2_ (n: 1357) *	96 ± 3 *88 ± 6*	96 ± 3 *89 ± 7*	95 ± 3 *85 ± 6*	0.013 *<0.001*
pH (*n*:200)	7.43 ± 0.05	7.44 ± 0.05	7.46 ± 0.08	0.047
PaO_2_ (*n*: 211)	82.1 ± 21.5	82.9 ± 21.2	73.4 ± 23.3	0.065
PaCO_2_ (*n*: 206)	36.8 ± 5.2	36.7 ± 5.0	37.7 ± 7.1	0.416
PaO_2_/FiO_2_ (*n*: 210)	328 ± 113	332 ± 113	287 ± 106	0.102
PaO_2_St (*n*: 206)	76.9 ± 21.6	77.7 ± 22.0	68.5 ± 15.4	0.084
PaO_2_St/FiO_2_ (*n*: 205) PaO_2_St/FiO_2_ (n: 1178) *	307 ± 108 *239 ± 107*	311 ± 110 *244 ± 108*	269 ± 82 *175 ± 98*	0.130 *<0.001*
**Imaging (%)**				
Single-sided ground glass thickening (*n*: 360)	14 (3.9%)	12 (3.7%)	4 (11.8%)	0.034
Bilateral ground glass thickening (*n*: 360)	275 (76.4%)	252 (77.3%)	23 (67.6%)	0.079
Unilateral parenchymal consolidation (*n*: 360)	30 (8.3%)	27 (8.3%)	3 (8.8%)	0.954
Bilateral parenchymal consolidation (*n*: 360)	69 (19.2%)	64 (19.6%)	5 (14.7%)	0.356
Unilateral pleural effusion (*n*: 360)	15 (4.2%)	12 (3.7%)	3 (8.8%)	0.034
Bilateral pleural effusion (*n*: 360)	30 (7.7%)	21 (6.4%)	9 (26.5%)	<0.001
**Hospital oxygen therapy (%)**				
Breathe in ambient air (*n*: 388)	220 (56.7%)	201 (57.8%)	19 (40.0%)	0.215
Nasal cannulas (*n*: 386)	65 (16.8%)	58 (16.8%)	7 (17.5%)	0.906
Facial mask (*n*: 386)	18 (4.7%)	14 (4.0%)	4 (10.0%)	0.091
Venturi mask (*n*: 386)	75 (19.4%)	66 (19.1%)	9 (22.5%)	0.604
NIV (*n*: 386)	2 (0.5%)	2 (0.6%)	0 (0%)	0.630
IMV (*n*: 388)	8 (2.1%)	7 (2.0%)	1 (2.1%)	0.837
**Clinical outcome (%)**				
Discharged home (*n*: 388)	261 (67.3%)	261 (75.0%)	0 (0%)	<0.001
Transferred to COVID-19 hotel (*n*: 388)	65 (16.8%)	65 (18.7%)	0 (0%)	0.003
Transferred to another department (*n*: 388)	22 (5.7%)	22 (6.3%)	0 (0%)	0.120

**Table 2:** Multivariable model for predicting death. Hb: hemoglobin; WBC: white blood cells; PLT: platelets; SpO_2_: oxygen saturation; In: natural logarithm.

Variables	B	Sig.	OR	95% CI for OR	
				Lower	Upper
Age (years)	0.116	0.000	1.124	1.07	1.18
Baseline SpO_2_ (%)	−0.175	0.026	0.840	0.72	0.98
Hb (g/dL)	−0.459	0.000	0.632	0.51	0.78
In [WBC (cell/µL)]	1.935	0.000	6.924	2.27	20.98
Neutrophils (%)	−0.045	0.051	0.956	0.913	1.00
In [PLT (cell/µL)]	−2.327	0.000	0.98	0.37	0.26
In [Creatinine (mg/dL)]	1.108	0.034	3.027	1.085	8.445
Constant	26.084	0.010			

**Table 3:** Comparative analysis of the ROC curve of our CZ-COVID-19 with the major COVID-19 scoring systems applied to the 388 patients in our derivation cohort.

Score	Year of Birth	Nation of Birth	External Validation of Score	Derivation Cohort (*n*)	Criteria for Score (*n*)	AUC *	95% CI *	Sensitivity (%) **	Specificity (%) **	AUC **	95% CI **	*p*-Value **	*p*-Value ***
CCI [10]	1987	USA	Yes	604	17	NA	NA	72.5	62.6	0.705	0.657–0.750	<0.001	<0.001
PSI [12]	1998	USA	Yes	38,000	20	NA	NA	99.9	52.4	0.884	0.804–0.879	<0.001	0.300
NEWS [11]	2012	UK	Yes	35,585	8	NA	NA	50.0	89.2	0.754	0.700–0.803	<0.001	0.011
ISARIC 4C [16]	2020	UK	Yes	66,705	11	0.790	0.780–0.790	82.5	65.8	0.771	0.726–0.812	<0.001	<0.001
HOME-CoV [17]	2020	FR	Yes	1696	7	0.876	0.847–0.906	95.0	42.5	0.710	0.677–0.768	<0.001	<0.001
ABC2-SPH [18]	2021	ES	Yes	3978	7	0.844	0.829–0.919	87.5	65.8	0.804	0.761–0.842	<0.001	0.014
CAPS-D [19]	2021	GER	Yes	1297	5	0.810	0.77–0.850	82.5	48.0	0.692	0.644–0.738	<0.001	0.007
SOARS [20]	2021	UK	Yes	983	5	0.820	NA	65.0	80.8	0.796	0.752–0.835	<0.001	<0.001
COVID-19 Sever Index **^$^** [21]	2021	ARG	Yes	220	16	0.940 ^$^	NA	80.0	55.5	0.755	0.709–0.797	<0.001	0.002
ASCL **^$$^** [22]	2022	ITA	Yes	390	11	0.713 ^$$^	NA	77.5	64.4	0.724	0.677–0.768	<0.001	<0.001
COEWS [23]	2023	m	Yes	3539	7	0.743	0.703–0.784	85.0	52.9	0.754	0.708–0.796	<0.001	0.001
NEWS 2 Plus [24]	2024	TH	?	725	10	0.798	0.767–0.830	70.8	80.0	0.815	0.765–0.858	<0.001	0.054
CZ COVID-19	2024	ITA	Yes	388	7	-	-	80.0	92.0	0.924	0.893–0.948	<0.001	-

95% CI, 95% confidence interval; ABC2-SPH, ABC2-SPH risk score; ARG, Argentina; ASCL, ASCL score abbreviated for age, sex; AUC, area under curve; CAPS-D, CAPS-D score; CCI, Charlson Comorbidity Index; COEWS, COVID-19 Early Warning Score; COVID-19 Sever Index, COVID-19 severity index; CRP at hospital admission, and LDH at hospital admission; CZ-COVID-19, Cascio-Zinna COVID-19-mortality Score; ES, Spain; FR, France; GER, Germany; HOME-CoV score, Hospitalization or Outpatient Management of patients with SARS-CoV-2 infection; ISARIC 4C, International Severe Acute Respiratory Infection Consortium–Coronavirus Clinical Characterisation Consortium; m, multicontinental retrospective study; *n*, absolute number; NA, not appropriated; NEWS 2 Plus, National Early Warning Score 2 Plus; NEWS, National Early Warning Score; PSI, Pneumonia Severity Index; SOARS, SOARS score abbreviated for SpO_2_, obesity, age, respiratory rate, stroke history; TH, Thailand; ITA, Italy; UK, United Kingdom; USA, United States of America. * In the original derivation cohort of COVID-19 patients. ** In our derivation cohort. *** Comparison of DeLong’s test *p*-value between reference CZ COVID-19 scores and the other scores. ^$^ The AUC value was obtained with data obtained 24 h before ICU transfer, not at hospital admission. ^$$^ The AUC value was obtained for the risk of a P/F ratio deterioration below 200, not for a risk of death.

## Data Availability

Data presented in this study are available on request from the corresponding author.

## Supplementary Materials

The following supporting information can be downloaded at: https://www.mdpi.com/article/10.3390/jcm13071832/s1, Spreadsheet A: CZ-COVID-19 Score calculator. Figure S1: Age pyramid in alive and dead patients. Derivation cohort, patients admitted to the University Hospital “Policlinico Paolo Giaccone” of Palermo (Italy) (a); external validation cohort, patients admitted to the Luigi Vanvitelli University Hospital of Campania (Italy) (b). Figure S2: Main comorbidities in patients who survived and who died. Table S1: Crude odd ratios (crude OR) and adjusted odds ratios (AOR) deriving from multiple logistic regression analysis. In bold are those associations that are statistically significant. pt.: patients; COPD: chronic obstructive pulmonary disease; TB: tuberculosis; s.f.: intravenous injection; HIV: human immunodeficiency virus; CKD: chronic kidney disease; AKI: acute kidney injury; CNS: central nervous system; PSI: Pneumonia Severity Index; CCI: Charlson Comorbidity Index; NEWS: National Early Warning Score; Hb: hemoglobin; WBC: white blood cells; PLT: platelets; LDH: lactate dehydrogenase; PT/INR: Prothrombin Time/International Normalized Ratio; aPTT: activated partial thromboplastin time; CRP: C-reactive protein; PCT: procalcitonin; IL-6: interleukin-6; BNPT: B-type natriuretic peptide; HR: heart rate; SpO_2_: oxygen saturation; Pa: arterial partial pressure; FiO_2_: fraction of inspired O_2_; PaO_2_St: standardized arterial partial pressure oxygen; NIV: non-invasive ventilation; IMV: invasive mechanical ventilation. Table S2: CZ-COVID-19 Score and associated score weights, in two patient cohorts. CZ-COVID-19 Score: Cascio-Zinna COVID-19-mortality Score.
