# Supplementary material for: COVID-19: The Development and Validation of a New Mortality Risk Score
Source: J Clin Med. 2024 Mar 22;13(7):1832. doi: 10.3390/jcm13071832 (PMC11012743; doi:10.3390/jcm13071832)
Supplement: Supplementary file 1 [file jcm-13-01832-s001.zip › Figure S1.pdf]

Color image

Figure S1. Age pyramid in alive and dead patients. Derivation cohort, patients admitted at the University Hospital "Policlinico Paolo Giaccone" of Palermo (Italy) (a); external validation cohort, patients admitted to the Luigi Vanvitelli University Hospital of Campania (Italy) (b).

(a

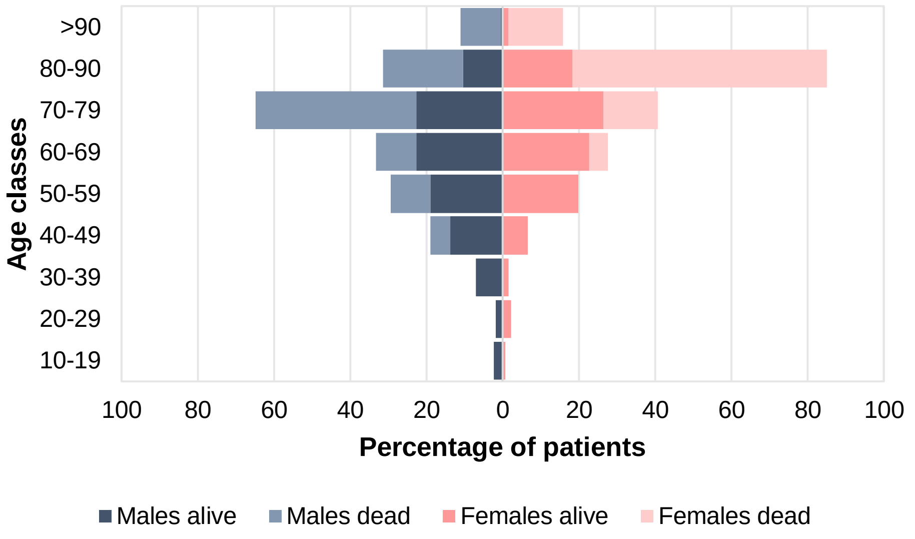
>90
80-90
70-79
60-69
50-59
40-49
30-39
20-29
10-19
Age classes
100 80 60 40 20 0 20 40 60 80 100
Percentage of patients
Males alive
Males dead
Females alive
Females dead

(b

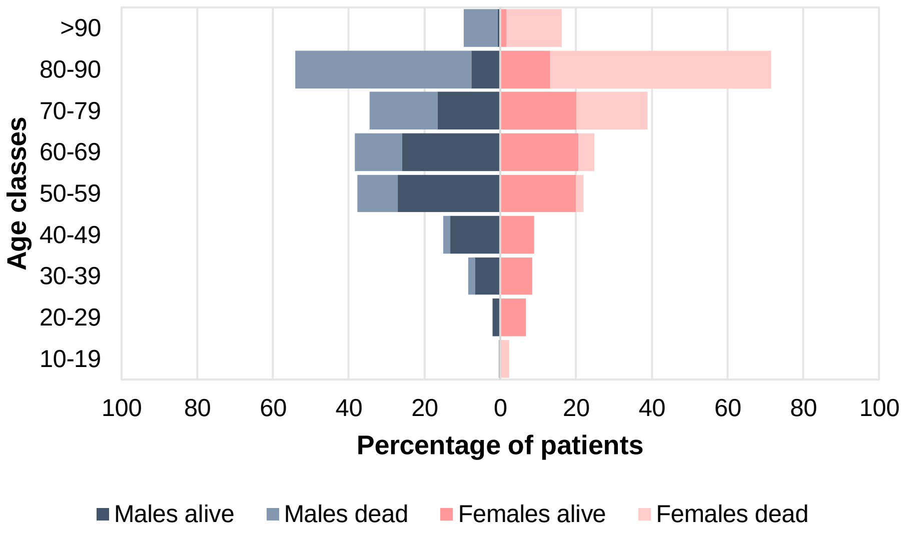
>90
80-90
70-79
60-69
50-59
40-49
30-39
20-29
10-19
Age classes
100 80 60 40 20 0 20 40 60 80 100
Percentage of patients
Males alive
Males dead
Females alive
Females dead
