# Supplementary material for: COVID-19: The Development and Validation of a New Mortality Risk Score
Source: J Clin Med. 2024 Mar 22;13(7):1832. doi: 10.3390/jcm13071832 (PMC11012743; doi:10.3390/jcm13071832)
Supplement: Supplementary file 1 [file jcm-13-01832-s001.zip › Figure S2.pdf]

**Color image**

**Figure S2.** Main comorbidities in patients who survived and who died.

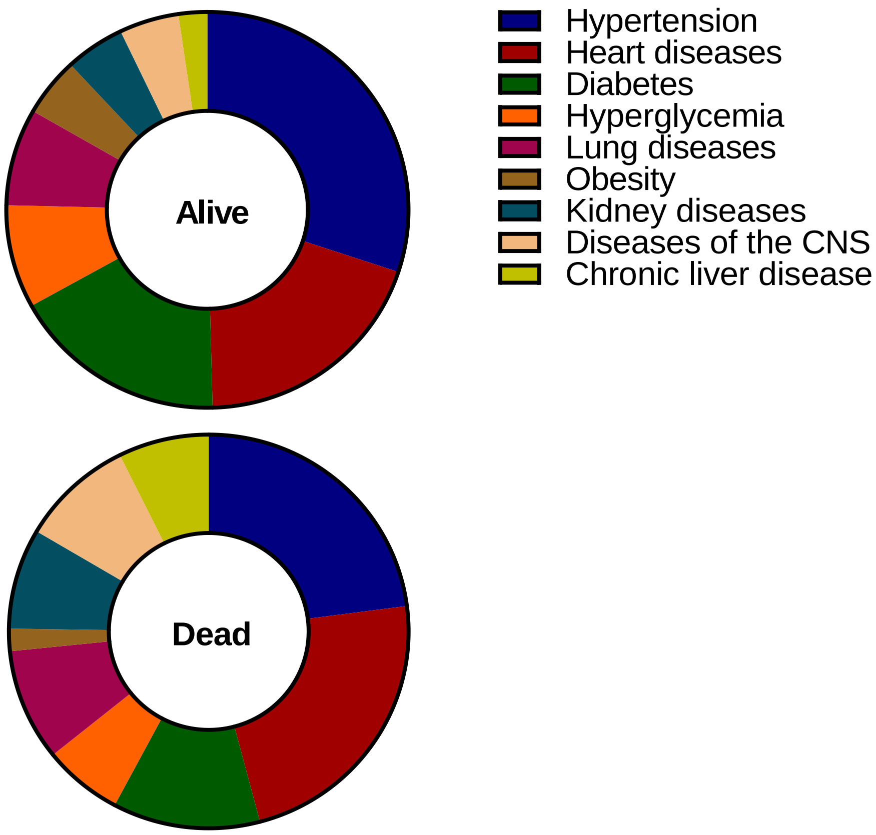
Alive
Dead
Hypertension
Heart diseases
Diabetes
Hyperglycemia
Lung diseases
Obesity
Kidney diseases
Diseases of the CNS
Chronic liver disease
