# Supplementary material for: COVID-19: The Development and Validation of a New Mortality Risk Score
Source: J Clin Med. 2024 Mar 22;13(7):1832. doi: 10.3390/jcm13071832 (PMC11012743; doi:10.3390/jcm13071832)
Supplement: Supplementary file 1 [file jcm-13-01832-s001.zip › Table S1.pdf]

Table S1. Crude odd ratios (crude OR) and adjusted odds ratios (AOR) deriving from multiple logistic regression analysis. In bold are those associations that are statistically significant. pt.: patients; COPD: chronic obstructive pulmonary disease; TB: tuberculosis; s.f.: intravenous injection; HIV: human immunodeficiency virus; CKD: chronic kidney disease; AKI: acute kidney injury; CNS: central nervous system; PSI: Pneumonia Severity Index; CCI: Charlson Comorbidity Index; NEWS: National Early Warning Score; Hb: haemoglobin; WBC: white blood cells; PLT: platelets; LDH: lactate dehydrogenase; PT/INR: Prothrombin Time/International Normalized Ratio; aPTT: activated partial thromboplastin time; CRP: C-reactive protein; PCT: procalcitonin; IL-6: interleukin-6; BNPT: B-type natriuretic peptide; HR: heart rate; SpO_2: oxygen saturation; Pa: arterial partial pressure; FiO_2: fraction of inspired O_2; PaO_2St: standardized arterial partial pressure oxygen; NIV: Non-invasive ventilation; IMV: invasive Mechanical Ventilation.

Variable (n)	All pt. (%)	Survivor (%)	Death (%)	Crude OR (CI95%)	AOR (CI95%)	P value
**Demographics**						
**Age (n: 388)**						
≤ 76 years	290 (74.7%)	279 (80.2%)	11 (27.5%)	1	1	**0.001**
> 76 years	98 (25.3%)	69 (1.8%)	29 (72.5%)	**10.66 (5.07-22.39)**	**7.21 (2.20-23.77)**	
**Sex (n: 388)**						
Male	231 (59.5%)	212 (60.9%)	19 (47.5%)	1		
Female	157 (40.5%)	136 (39.1%)	21 (52.5%)	1.72 (0.89-3.32)		
**Age-adjusted for sex (n: 388)**						
Sex M≤72 or F≤76	261 (67.3%)	253 (72.2%)	8 (20.0%)	1	1	**0.003**
Sex M>72 of F>76	127 (32.7%)	95 (27.3%)	32 (80.0%)	**10.65 (4.74-23.94)**	**6.24 (1.84-21.13)**	
**Comorbidities**						
**Number of comorbidities (n: 388)**						
0	39 (10.1%)	39 (11.2%)	0 (0%)	0	0	
1-2	119 (30.7%)	113 (32.5%)	6 (15.0%)	0.37 (0.15-0.90)	1	
3-4	126 (32.5%)	114 (32.8%)	12 (30.0%)	0.88 (0.43-1.79)	1	
>4	104 (26.8%)	82 (23.6%)	22 (55.0%)	**3.97 (2.03-7.75)**	1.38 (0.38-5.08)	0.627
**Mute past medical history (n: 388)**						
Negative	349 (88.9%)	309 (88.8%)	40 (100%)	1		
Positive	39 (10.1%)	39 (11.2%)	0 (0%)	0		
**Obesity (n: 388)**						
Negative	357 (92.0%)	319 (91.7%)	38 (95.0%)	1		
Positive	31 (8.0%)	29 (8.3%)	2 (5.0%)	0.58 (0.13-2.52)		
**Diabetes (n: 388)**						
Negative	270 (69.6%)	243 (69.8%)	27 (67.5%)	1		
Positive	118 (30.4%)	105 (30.2%)	13 (32.5%)	1.14 (0.55-2.24)		
**Hypertension (n: 388)**						
Negative	181 (46.6%)	166 (47.7%)	15 (37.5%)	1		
Positive	207 (53.4%)	182 (52.3%)	25 (62.5%)	1.52 (0.77-2.98)		
**COLD (n: 388)**						
Negative	359 (92.5%)	324 (93.1%)	35 (87.5%)	1		
Positive	29 (7.5%)	24 (6.9%)	5 (12.5%)	1.93 (0.69-5.37)		
**Asthma (n: 388)**						
Negative	382 (98.5%)	342 (98.3%)	40 (100%)	1		
Positive	6 (1.5%)	6 (1.7%)	0 (0%)	0		
**TB (n: 388)**				1		
Negative	385 (99.2%)	346 (99.4%)	39 (97.5%)	4.43 (0.39-50.04)		
Positive	3 (0.8%)	2 (0.6%)	1 (2.5%)			
**Other lung diseases (n: 388)**						
Negative	397 (94.6%)	332 (95.4%)	35 (87.5%)	1	1	0.652
Positive	21 (5.4%)	16 (4.6%)	5 (12.5%)	**2.96 (1.02-8.58)**	1.60 (0.21-12.42)	
**Active smoking (n: 388)**						
Negative	375 (96.6%)	337 (96.8%)	38 (95.0%)	1		
Positive	13 (3.4%)	11 (3.2%)	2 (5.0%)	1.61 (0.34-7.54)		
**Drugs s.f. (n: 388)**						
Negative	387 (99.7%)	347 (99.7%)	40 (100%)	1		
Positive	1 (0.3%)	1 (0.3%)	0	0		
**Alcoholism (n: 388)**						
Negative	384 (99.0%)	344 (98.9%)	40 (100%)	1		
Positive	4 (1.0%)	4 (1.1%)	0 (0%)	0		
**Depression (n: 388)**						
Negative	371 (95.6%)	331 (95.1%)	40 (100%)	1		
Positive	17 (4.4%)	17 (4.9%)	0 (0%)	0		
**Suicidal ideation (n: 388)**						
Negative	387 (99.7%)	347 (99.7%)	40 (100%)	1		
Positive	1 (0.3%)	1 (0.3%)	0	0		
**Hepatosplenomegaly (n: 388)**						
Negative	385 (99.2%)	346 (99.4%)	39 (97.5%)	1		
Positive	3 (0.8%)	20 (0.6%)	1 (2.5%)	4.44 (0.39-50.04)		
**Gastrointestinal bleeding (n: 388)**						
Negative	386 (99.5%)	346 (99.4%)	40 (100%)	1		

Positive	2 (0.5%)	2 (0.6%)	0 (0%)	0		
**Hyperglycemia (n: 388)**						
<140 mg/dl	330 (85.1%)	297 (85.3%)	33 (82.5%)	1		
140-180 mg/dl	28 (7.2%)	23 (6.6%)	5 (12.5%)	2.01 (0.72-5.63)		
>180 mg/dl	30 (7.7%)	28 (8.0%)	2 (5.0%)	0.60 (0.14-2.62)		
**HIV+ (n: 388)**						
Negative	385 (99.2%)	346 (99.4%)	39 (97.5%)	1		
Positive	3 (0.8%)	2 (0.6%)	1 (2.5%)	4.44 (0.40-50.04)		
**Chronic liver disease (n: 388)**						
Negative	366 (94.3%)	334 (96.0%)	32 (80.0%)	1	1	0.172
Positive	22 (5.7%)	14 (4.0%)	8 (20.0%)	**6.96 (2.33-15.29)**	3.49 (0.58-20.93)	
**Cardiovascular disease (n: 388)**						
Negative	306 (78.9%)	282 (81.0%)	24 (60.0%)	1	1	0.617
Positive	82 (21.1%)	66 (19.0%)	16 (40.0%)	**2.85 (1.43-5.66)**	1.35 (0.42-4.39)	
**Other heart conditions (n: 388)**						
Negative	327 (84.3%)	296 (85.1%)	31 (77.5%)	1		
Positive	61 (15.7%)	52 (14.9%)	9 (22.5%)	1.65 (0.74-3.67)		
**CKD (n: 388)**						
Negative	356 (91.8%)	324 (93.1%)	32 (80.0%)	1	1	0.114
Positive	32 (8.2%)	24 (6.9%)	8 (20.0%)	**3.37 (1.40-8.13)**	0.25 (0.04-1.41)	
**AKI (n: 388)**						
Negative	383 (98.7%)	344 (98.9%)	39 (97.5%)	1		
Positive	5 (1.3%)	4 (1.1%)	1 (2.5%)	2.21 (0.24-20.22)		
**Hemodialysis (n: 388)**						
Negative	387 (99.7%)	347 (99.7%)	40 (100%)	1		
Positive	1 (0.3%)	1 (0.3%)	0 (0%)	0		
**Disease of the CNS (n: 388)**						
Negative	349 (89.9%)	319 (91.7%)	30 (75.0%)	1	1	**0.050**
Positive	39 (10.1%)	29 (8.3%)	10 (25.0%)	**3.66 (1.63-8.25)**	**4.15 (1.00-17.18)**	
**Organ transplant (n: 388)**						
Negative	383 (98.6%)	343 (98.6%)	40 (100%)	1		
Positive	5 (1.4%)	5 (1.4%)	0 (0%)	0		
**Other comorbidities (n: 388)**						
Negative	135 (34.8%)	127 (36.5%)	8 (20.0%)	1	1	0.451
Positive	253 (65.2%)	221 (63.5%)	32 (80.0%)	**2.29 (1.08-5.14)**	1.74 (0.41-7.32)	
**Time passed**						
**Days of hospitalization (n: 386)**						
≤ 13 days	223 (57.8%)	213 (61.6%)	10 (25.0%)	1		
> 13 days	163 (42.2%)	133 (38.4%)	30 (75.0%)	**4.81 (2.28-10.15)**		
**Days from symptom onset to hospitalization (n: 309)**						
> 4 days	193 (62.5%)	184 (65.7%)	9 (31.0%)	1	1	0.172
≤ 4 days	116 (37.5%)	96 (34.3%)	20 (69.0%)	**4.26 (1.87-9.72)**	2.19 (0.71-6.95)	
**Days from positive swab to hospitalization (n: 360)**						
> 8 days	43 (11.9%)	41 (12.7%)	2 (5.6%)	1		
≤ 8 days	317 (88.1%)	283 (87.3%)	34 (94.4%)	2.46 (0.57-10.64)		
**Days from symptom onset to positive swab (n: 302)**						
> 4 days	123 (40.7%)	119 (43.4%)	4 (14.3%)	1	1	**0.025**
≤ 4 days	179 (59.3%)	155 (56.6%)	24 (85.7%)	**4.61 (1.56-13.64)**	**5.38 (1.23-23.55)**	
**Symptoms clinical onset**						
**Fever (n: 388)**						
Negative	187 (48.2%)	165 (47.4%)	22 (55.0%)	1		
Positive	201 (51.8%)	183 (52.6%)	18 (45.0%)	0.74 (0.38-1.43)		
**Cough (n: 388)**						
Negative	293 (75.5%)	255 (73.3%)	38 (95.0%)	1		
Positive	95 (24.5%)	93 (26.7%)	2 (5.0%)	0.14 (0.03-0.61)		

**Sputum (n: 388)**						
Negative	384 (99.0%)	345 (99.1%)	39 (97.5%)	1		
Positive	4 (1.0%)	3 (0.9%)	1 (2.5%)	2.95 (0.30-29.04)		
**Asthenia (n: 388)**						
Negative	317 (81.7%)	284 (81.6%)	33 (82.5%)	1		
Positive	71 (18.3%)	64 (18.4%)	7 (17.5%)	0.94 (0.34-2.23)		
**Dyspnea (n: 388)**						
Negative	244 (62.9%)	219 (62.9%)	25 (62.5%)	1		
Positive	144 (37.1%)	129 (37.1%)	15 (37.5%)	1.02 (0.52-2.00)		
**Anorexia (n: 388)**						
Negative	384 (99.0%)	344 (98.9%)	40 (100.0%)	1		
Positive	4 (1.0%)	4 (1.1.%)	0 (0%)	0		
**Myalgia (n: 388)**						
Negative	364 (93.8%)	326 (93.7%)	38 (95.0%)	1		
Positive	24 (6.2%)	22 (6.3%)	2 (5.0%)	0.78 (0.18-3.48)		
**Arthralgia (n: 388)**						
Negative	363 (93.6%)	324 (93.1%)	39 (93.6%)	1		
Positive	25 (6.4%)	24 (6.9%)	1 (2.5%)	0.35 (0.05-2.63)		
**Loss of smell (n: 388)**						
Negative	375 (96.6%)	335 (96.3%)	40 (100%)	1		
Positive	13 (3.4%)	13 (96.3%)	0 (0%)	0		
**Loss of taste (n: 388)**						
Negative	375 (96.6%)	335 (96.3%)	40 (100.0%)	1		
Positive	13 (3.4%)	13 (96.3%)	0 (0%)	0		
**Diarrhea (n: 388)**						
Negative	354 (91.2%)	315 (90.5%)	39 (97.5%)	1		
Positive	34 (8.8%)	33 (9.5%)	1 (2.5%)	0.25 (0.03-1.84)		
**Vomit (n: 388)**						
Negative	370 (95.4%)	332 (95.4%)	38 (95.0%)	1		
Positive	18 (4.6%)	16 (4.6%)	2 (5.0%)	1.09 (0.24-4.93)		
**Headache (n: 388)**						
Negative	361 (93.0%)	321 (92.2%)	40 (100%)	1		
Positive	27 (7.0%)	27 (7.8%)	0 (0%)	0		
**Chest pain (n: 388)**						
Negative	369 (95.1%)	330 (94.8%)	39 (97.5%)	1		
Positive	19 (4.9%)	18 (5.2%)	1 (2.5%)	0.47 (0.06-3.62)		
**Abdominal pain (n: 388)**						
Negative	366 (94.3%)	326 (93.7%)	40 (100%)	1		
Positive	22 (5.7%)	22 (6.3%)	0 (0%)	0		
**Gastrointestinal bleeding (n: 388)**						
Negative	386 (99.5%)	346 (99.4%)	40 (100%)	1		
Positive	2 (0.5%)	2 (0.6%)	0 (%)	0		
**Other symptoms (n: 387)**						
Negative	299 (77.3%)	270 (77.8%)	29 (72.5%)	1		
Positive	88 (22.7%)	77 (22.2%)	11 (27.5%)	1.33 (0.64-2.78)		
**Score**						
**PSI (n: 388)**						
Class I-II	190 (49.0%)	189 (54.3%)	1 (2.5%)	1	1	**0.038**
Class III-IV-V	198 (51.0%)	159 (45.7%)	39 (97.5%)	**46.36 (6.30-341.22)**	**19.33 (1.18-317.77)**	
**CCI (n: 388)**						
Class 0	22 (5.6%)	22 (6.2%)	0 (0%)	-	-	
Class 1-2	86 (22.2%)	83 (23.9%)	3 (7.5%)	0.26 (0.08-0.86)	1	
Class 3-4	121 (31.2%)	113 (32.5%)	8 (20.0%)	0.52 (0.23-1.16)	1	
Class >4	159 (41.0%)	130 (37.4%)	29 (72.5%)	**4.42 (2.14-9.15)**	0.83 (0.23-2.98)	0.779
**NEWS (n: 284)**						
Class 0	37 (13.0%)	37 (14.2%)	0 (0%)	-		
Class 1-4	166 (58.5%)	156 (60.0%)	10 (41.7%)	0.47 (0.20-1.11)		
Class 5-6	41 (14.4%)	39 (15.0%)	2 (8.3%)	0.52 (0.12-2.28)		
Class ≥7	40 (14.1%)	28 (10.8%)	12 (50.0%)	**8.29 (3.40-20.20)**		

**PSI and CCIS (n: 388)**						
PSI I-III and CCI 0	22 (6.3%)	22 (7.9%)	0 (0%)	0	-	
PSI I-III and CCI 1-2	84 (21.6%)	83 (23.9%)	1 (2.5%)	0.08 (0.11-0.61)	1	
PSI I-III and CCI 3-4	105 (27.1%)	100 (28.7%)	5 (12.5%)	0.35 (0.14-0.93)	1	
PSI I-II-III and CCI >4	81 (20.9%)	75 (21.6%)	6 (15.0%)	0.64 (0.26-1.59)	1	
PSI IV-V and CCI 0	0 (0%)	0 (0%)	0 (0%)	0	-	
PSI IV-V and CCI 1-2	2 (2.1%)	0 (0%)	2 (5.0%)	-	-	
PSI IV-V and CCI 3-4	15 (3.9%)	12 (3.4%)	3 (7.5%)	2.27 (0.62-8.42)	1.73 (0.19-15.58)	0.624
PSI IV-V and CCI >4	78 (20.1%)	55 (15.8%)	23 (57.5%)	**7.21 (3.62-14.37)**	2.94 (0.97-8.97)	0.058
**Laboratory tests**						
**Hb (n: 386)**						
> 12.1 g/dL	249 (64.5%)	238 (68.6%)	11 (28.2%)	1	1	**0.016**
≤ 12.1 g/dL	137 (35.5%)	109 (31.4%)	28 (71.8%)	**5.57 (2.67-11.58)**	**4.19 (1.30-13.49)**	
**Hb adjusted for sex (n: 388)**						
Hb M>11.6 or F>12.1	263 (67.8%)	251 (72.1%)	12 (30.0%)	1	1	**0.001**
Hb M≤11.6 or F≤12.1	125 (32.2%)	97 (27.9%)	28 (70.0%)	**6.038 (2.95-12.35)**	**9.48 (2.53-35.67)**	
**WBC (n: 387)**						
≤ 8,700 cell/μL	248 (64.1%)	232 (66.9%)	16 (40.0%)	1	1	
> 8,700 cell/μL	139 (35.9%)	115 (33.1%)	24 (60.0%)	**3.03 (1.55-5.92)**	**4.02 (1.32-12.21)**	**0.014**
WBC (n: 1357)						
**Number of neutrophils (n: 381)**						
≤ 7,547 cell/μL	253 (66.4%)	238 (69.4%)	15 (39.5%)	1	1	**0.005**
> 7,547 cell/μL	128 (33.6%)	105 (30.6%)	23 (60.5%)	**3.47 (1.74-6.93)**	**5.38 (1.66-17.48)**	
**% neutrophils (n: 381)**						
≤ 83.6 %	182 (47.8%)	174 (50.7%)	8 (21.1%)	1	1	0.077
> 83.6 %	199 (52.2%)	169 (49.3%)	30 (79.8%)	**3.86 (1.72-8.66)**	2.77 (0.90-8.57)	
**Number of lymphocytes (n: 381)**						
> 1,261 cell/μL	156 (40.9%)	147 (42.9%)	9 (23.7%)	1	1	0.503
≤ 1,261 cell/μL	225 (59.1%)	196 (57.1%)	29 (76.3%)	**2.42 (1.11-5.26)**	0.67 (0.21-2.16)	
**% lymphocytes (n: 381)**						
> 16.3 %	182 (47.8%)	174 (50.7%)	8 (21.1%)	1	1	0.092
≤ 16.3 %	199 (52.2%)	169 (49.3%)	30 (78.9%)	**3.86 (1.72-8.66)**	2.63 (0.86-8.07)	
**PLT (n: 383)**						
> 147 x10^3/μL	328 (85.6%)	302 (88.0%)	26 (65.0%)	1	1	**0.002**
≤ 147 x10^3/μL	55 (14.4%)	41 (12.0%)	14 (35.0%)	**3.97 (1.92-8.20)**	**7.57 (2.14-26.79)**	
**Creatinine (n: 388)**						
≤ 1.05 mg/dL	345 (88.9%)	319 (91.7%)	26 (65.0%)	1	1	**0.013**
> 1.05 mg/dL	43 (11.3%)	29 (8.3%)	14 (35.0%)	**6.38 (2.54-16.01)**	**6.28 (1.47-26.84)**	
**LDH (n: 261)**						
≤ 331 U/L	196 (75.1%)	186 (77.2%)	10 (50.0%)	1		
> 331 U/L	65 (24.9%)	55 (22.8%)	10 (50.0%)	**3.38 (1.34-8.54)**		
**PT/INR (n: 356)**						
≤ 1.18	245 (68.8%)	227 (70.3%)	18 (54.5%)	1		
> 1.18	111 (31.2%)	96 (29.7%)	15 (45.5%)	1.97 (0.95-4.07)		
**aPTT (n: 246)**						
≤ 28.4 seconds	133 (54.1%)	126 (55.5%)	7 (36.8%)	1		
> 28.4 seconds	113 (45.9%)	101 (44.5%)	12 (63.2%)	2.14 (0.81-5.63)		
**Fibrinogen (n: 308)**						
≤ 378 mg/dL	233 (75.6%)	217 (77.2%)	16 (59.3%)	1	1	0.434
> 378 mg/dL	75 (24.4%)	64 (22.8%)	11 (40.7%)	**2.33 (1.030-5.27)**	1.56 (0.51-4.75)	
**D-dimer (n: 319)**						
≤ 941 ng/mL EFU	186 (58.3%)	178 (61.4%)	8 (27.6%)	1	1	0.476
> 941 ng/mL EFU	133 (41.7%)	112 (38.6%)	21 (72.4%)	**4.17 (1.78-9.74)**	1.60 (0.44-5.87)	
**CRP (n: 356)**						
≤ 79.8 mg/L	277 (77.8%)	257 (79.3%)	20 (62.5%)	1	1	0.649
> 79.8 mg/L	79 (22.2%)	67 (20.7%)	12 (37.5%)	**2.30 (1.07-4.94)**	0.77 (0.25-2.37)	
**PCT (n: 196)**						
≤ 0.14 μg/L	126 (64.3%)	116 (67.4%)	10 (41.7%)	1		
> 0.14 μg/L	70 (35.7%)	56 (32.6%)	14 (58.3%)	**2.90 (1.21-6.93)**		
**IL-6 (n: 232)**						
≤ 36.1 pg/mL	155 (66.8%)	146 (69.9%)	9 (39.1%)	1		
> 36.1 pg/mL	77 (33.2%)	63 (30.1%)	14 (60.9%)	**3.61 (1.48-8.76)**		
**Triglycerides (n: 97)**						
≤ 155 mg/dL	75 (77.3%)	65 (74.7%)	10 (100%)	1		
> 155 mg/dL	22 (22.7%)	22 (25.3%)	0 (0%)	0		

**Ferritin (n: 111)**						
≤ 1,409 ng/mL	94 (84.7%)	87 (87.9%)	7 (58.3%)	1		
> 1,409 ng/mL	17 (15.3%)	15 (12.1%)	5 (41.7%)	**5.18 (1.42-18.94)**		
**Troponin (ng/L) (n: 56)**						
> 7.2 mg/L	42 (75.0%)	40 (80.0%)	2 (33.3%)	1		
≤ 7.2 mg/L	14 (25.0%)	10 (20.0%)	4 (66.7%)	**8.0 (1.27-50.04)**		
**BNPT (n: 58)**						
> 19.51 pg/mL	52 (89.7%)	48 (94.1%)	4 (57.1%)	1		
≤ 19.51 pg/mL	6 (10.3%)	3 (5.9%)	3 (42.9%)	**12.0 (1.79-80.04)**		
**Respiratory function**						
**Acts breath/minute (n: 140)**						
≤ 23 acts/minute	111 (79.3%)	105 (83.3%)	6 (42.9%)	1		
> 23 acts/minute	29 (20.7%)	21 (16.7%)	8 (57.1%)	**6.67 (2.09-21.22)**		
**HR (n: 287)**						
≤ 81 acts/minute	140 (48.8%)	131 (50.0%)	9 (36.0%)	1		
> 81 acts/minute	147 (51.2%)	131 (50.0%)	16 (64.4%)	1.79 (0.76-4.17)		
**Baseline SpO_2 (n: 361)**						
> 95 %	227 (62.9%)	212 (64.8%)	15 (44.1%)	1	1	**0.009**
≤ 95 %	134 (37.1%)	115 (35.2%)	19 (55.9%)	**2.34 (1.14-4.77)**	**4.96 (1.48-16.62)**	
**pH (n: 200)**						
≤ 7.4	46 (23.0%)	44 (24.2%)	2 (10.5%)	1		
> 7.4	154 (77.0%)	137 (75.7%)	17 (89.5%)	2.73 (0.61-12.28)		
**PaO_2 (n: 211)**						
> 64	184 (87.2%)	173 (90.1%)	11 (57.9%)	1		
≤ 64	27 (12.8%)	19 (9.9%)	8 (42.1%)	**6.62 (2.37-18.48)**		
**$PaCO_2$ (n: 206)**						
≤ 37.3	127 (61.7%)	120 (63.8%)	7 (38.9%)	1		
> 37.3	79 (38.3%)	68 (36.2%)	11 (61.1%)	**2.77 (1.03-7.49)**		
**PaO_2/FiO_2 (n: 210)**						
> 300	128 (61.0%)	121 (63.0%)	7 (38.9%)	1		
≤ 300	82 (39.0%)	71 (37.0%)	11 (61.1%)	2.67 (0.99-7.22)		
**PaO_2St (n: 206)**						
> 78	83 (40.3%)	81 (43.1%)	2 (11.1%)	1		
≤ 78	123 (59.7%)	107 (56.9%)	16 (88.9%)	**6.07 (1.35-27.09)**		
**PaO_2St/FiO_2 (n: 205)**						
> 187	176 (85.9%)	165 (87.8%)	11 (64.7%)	1		
≤ 187	29 (14.1%)	23 (12.2%)	6 (35.3%)	**3.92 (1.32-11.59)**		
**Imaging**						
**Single-sided ground glass thickening (n: 360)**						
Negative	344 (95.6%)	314 (96.3%)	30 (88.2%)	1	1	
Positive	14 (3.9%)	12 (3.7%)	4 (11.8%)	**3.49 (1.059-11.49)**	2.98 (0.33-27.01)	0.333
**Bilateral ground glass thickening (n: 360)**						
Negative	85 (23.6%)	74 (22.7%)	11 (32.4%)	1		
Positive	275 (76.4%)	252 (77.3%)	23 (67.6%)	0.61 (0.28-1.32)		
**Unilateral parenchymal consolidation (n: 360)**						
Negative	330 (91.7%)	299 (91.7%)	31 (91.2%)	1		
Positive	30 (8.3%)	27 (8.3%)	3 (8.8%)	1.07 (0.31-3.74)		
**Bilateral parenchymal consolidation (n: 360)**						
Negative	291 (80.8%)	262 (80.4%)	29 (85.3%)	1		
Positive	69 (19.2%)	64 (19.6%)	5 (14.7%)	0.71 (0.24-1.90)		
**Unilateral pleural effusion (n: 360)**						
Negative	245 (95.8%)	314 (96.3%)	31 (91.2%)	1		
Positive	15 (4.2%)	12 (3.7%)	3 (8.8%)	2.53 (0.68-9.46)		
**Bilateral pleural effusion (n: 360)**						
Negative	330 (91.7%)	305 (93.6%)	25 (73.5%)	1	1	
Positive	30 (8.3%)	21 (6.4%)	9 (26.5%)	**5.23 (2.17-12.62)**	3.42 (0.80-14.60)	0.097
Hospital oxygen therapy						

**Breathe in ambient air (n: 388)**				
Negative	168 (43.3%)	147 (42.2%)	21 (52.5%)	1
Positive	220 (56.7%)	201 (57.8%)	19 (47.5%)	0.66 (0.34-1.27)
**Nasal cannulas (n: 386)**	321 (83.2%)	288 (83.2%)	33 (82.5%)	1
Negative	65 (16.8%)	58 (16.8%)	7 (17.5%)	1.05 (0.44-2.50)
Positive				
**Facial mask (n: 386)**				
Negative	368 (95.3%)	332 (96.0%)	36 (90.0%)	1
Positive	18 (4.7%)	14 (4.0%)	4 (10.0%)	2.64 (0.83-8.43)
**Venturi mask (n: 386)**				
Negative	311 (80.6%)	280 (90.9%)	31 (77.5%)	1
Positive	75 (19.4%)	66 (19.1%)	9 (22.5%)	1.23 (0.56-2.71)
**NIV (n: 386)**				
Negative	384 (99.5%)	344 (99.4%)	40 (100%)	1
Positive	2 (0.5%)	2 (0.6%)	0 (0%)	0
**IMV (n: 388)**				
Negative	380 (97.9%)	341 (98.0%)	39 (97.5%)	1
Positive	8 (2.1%)	7 (2.0%)	1 (2.5%)	1.25 (0.15-10.42)
**Clinical outcome**				
**Discharged home (n: 388)**				
Negative	127 (32.7%)	87 (25.0%)	40 (100%)	1
Positive	261 (67.3%)	261 (75.0%)	0 (0%)	0
**Transferred to Covid Hotel (n: 388)**				
Negative	323 (83.2%)	283 (81.3%)	40 (100%)	1
Positive	65 (16.8%)	65 (18.7%)	0 (0%)	0
**Transferred to another department (n: 388)**				
Negative	366 (94.3%)	326 (93.7%)	40 (100%)	1
Positive	22 (5.7%)	22 (6.3%)	0 (0%)	0
