# Supplementary material for: COVID-19: The Development and Validation of a New Mortality Risk Score
Source: J Clin Med. 2024 Mar 22;13(7):1832. doi: 10.3390/jcm13071832 (PMC11012743; doi:10.3390/jcm13071832)
Supplement: Supplementary file 1 [file jcm-13-01832-s001.zip › Table S2.pdf]

<u>Color image</u>

Table S2. CZ-COVID-19 Score and associated score weights, in two patient cohorts. CZ-COVID-19 Score: Cascio-Zinna COVID-19-mortality Score.

CZ Covid-19	Derivation cohort (n:388)			External validation cohort (n:1753)			Risk category	Risk score	Mortality
	All	Alive	Dead	All	Alive	Dead			
**-10**	-	-	-	2 (0.1%)	2 (0.1%)	0 (0%)			
**-9**	-	-	-	2 (0.1%)	1 (0.1%)	1 (1.0%)			
**-8**	-	-	-	7 (0.5%)	4 (0.3%)	3 (2.9%)			
**-7**	-	-	-	11 (0.8%)	7 (0.3%)	4 (3.8%)			
**-6**	1 (0.3%)	0 (0%)	1 (2.5%)	26 (1.9%)	16 (1.3%)	10 (9.6%)			
**-5**	-	-	-	39 (2.9%)	29 (2.2%)	11 (10.6%)	High	≤ 1	80%
**-4**	1 (0.3%)	0 (0%)	1 (2.5%)	61 (4.5%)	51 (4.1%)	10 (9.6%)			
**-3**	-	-	-	94 (6.9%)	75 (6.0%)	19 (18.3%)			
**-2**	4 (1.0%)	1 (0.3%)	3 (7.5%)	122 (9.0%)	103 (8.3%)	19 (18.3%)			
**-1**	13 (3.4%)	3 (0.9%)	10 (25.0%)	151 (11.1%)	141(11.3%)	10 (9.6%)			
**0**	13 (3.4%)	6 (1.7%)	7 (17.5%)	158 (11.6%)	152(12.1%)	6 (5.8%)			
**1**	32 (8.2%)	22 (6.3%)	10 (25.5%)	167 (12.3%)	161(12.8%)	6 (5.8%)			
**2**	52 (13.4%)	49 (14.1%)	3 (7.5%)	142 (10.5%)	139(11.1%)	3 (2.9%)	Moderate	2-3	15%
**3**	53 (13.7%)	50 (14.4%)	3 (7.5%)	103 (7.6%)	102 (8.1%)	1 (1.0%)			
**4**	56 (14.4%)	55 (15.8%)	1 (2.5%)	84 (6.2%)	83 (6.6%)	1 (1.0%)			
**5**	49 (12.6%)	48 (13.8%)	1 (2.5%)	59 (4.3%)	59 (4.7%)	0 (0%)			
**6**	38 (9.8%)	38 (10.8%)	0 (0%)	45 (3.3%)	45 (3.6%)	0 (0%)			
**7**	36 (9.3%)	36 (10.3%)	0 (0%)	54 (4.0%)	54 (4.3%)	0 (0%)	Low	≥ 4	5%
**8**	17 (4.4%)	17 (4.9%)	0 (0%)	17 (1.3%)	17 (1.3%)	0 (0%)			
**9**	14 (3.6%)	14 (4.0%)	0 (0%)	12 (0.9%)	12 (1.0%)	0 (0%)			
**10**	5 (1.3%)	5 (1.4%)	0 (0%)	-	-	-			
**11**	4 (1.0%)	4 (1.1%)	0 (0%)	1 (0.1%)	1 (0.1%)	0 (0%)			
